# Poor Pre-operative Nutritional Status Is a Risk Factor of Post-operative Infections in Patients With Gastrointestinal Cancer—A Multicenter Prospective Cohort Study

**DOI:** 10.3389/fnut.2022.850063

**Published:** 2022-05-27

**Authors:** Li Zhang, Siwen Wang, Xuejin Gao, Tingting Gao, Lingli Huang, Bo Lian, Yingchao Gu, Jianjiao Chen, Dong Guo, Zhenyi Jia, Yong Wang, Fangyou Gong, Junde Zhou, Zhigang Xue, Zhida Chen, Jielian Xu, Leilei Wang, Jun Qian, Guifang Deng, Hao Hu, Yao Nie, Gang Li, Mengbin Li, Hua Yang, Wei Zhao, Yanbing Zhou, Huanlong Qin, Xiaoting Wu, Kunhua Wang, Qiang Chi, Jianchun Yu, Yun Tang, Pianhong Zhang, Gang Jin, Bin Ouyang, Guoli Li, Dong Hang, Xinying Wang

**Affiliations:** ^1^Department of General Surgery, Jinling Hospital, Medical School of Nanjing University, Nanjing, China; ^2^Department of General Surgery, Jinling Hospital, Southern Medical University, Nanjing, China; ^3^Department of General Surgery, Cancer Institute of Jiangsu Province, Jiangsu Cancer Hospital, The Affiliated Cancer Hospital of Nanjing Medical University, Nanjing, China; ^4^Department of Gastrointestinal Surgery, The First Affiliated Hospital of Air Force Medical University, Xi'an, China; ^5^Department of General Surgery, The Second Affiliated Hospital, Army Medical University, Chongqing, China; ^6^Department of General Surgery, Northern Jiangsu Province People's Hospital, Yangzhou, China; ^7^Department of Gastrointestinal Surgery, The Affiliated Hospital of Qingdao University, Qingdao, China; ^8^Department of General Surgery, Shanghai Tenth People's Hospital, School of Medicine, Tongji University, Shanghai, China; ^9^Department of Gastrointestinal Surgery, West China Hospital, Sichuan University, Chengdu, China; ^10^Department of General Surgery, First Affiliated Hospital of Kunming Medical University, Kunming, China; ^11^Department of General Surgery, The 2nd Affiliated Hospital of Harbin Medical University, Ha'erbin, China; ^12^Department of General Surgery, Peking Union Medical College Hospital, Chinese Academy of Medical Sciences, Beijing, China; ^13^Department of General Surgery, First Medical Center of Chinese PLA General Hospital, Beijing, China; ^14^Department of Clinical Nutrition, The Affiliated Jiangning Hospital of Nanjing Medical University, Nanjing, China; ^15^Department of Clinical Nutrition, The Second Affiliated Hospital, Zhejiang University School of Medicine, Hangzhou, China; ^16^Department of Gastrointestinal Surgery, Changzhou Second People's Hospital, Nanjing Medical University, Changzhou, China; ^17^Department of Clinical Nutrition, Union Shenzhen Hospital of Huazhong University of Science and Technology, Shenzhen, China; ^18^Department of Hepatobiliary Pancreatic Surgery, Changhai Hospital, The Second Military Medical University, Shanghai, China; ^19^Department of Critical Care Medicine, The First Affiliated Hospital of Sun Yat-sen University, Guangzhou, China; ^20^Jiangsu Key Lab of Cancer Biomarkers, Prevention and Treatment, Department of Epidemiology, Nanjing Medical University, Nanjing, China

**Keywords:** NRS2002, PG-SGA, gastric cancer, colorectal cancer (CRC), post-operation infection

## Abstract

**Objective:**

This study aimed to assess the prognostic value of the Nutritional Risk Score 2002 (NRS2002) and patient-generated subjective global assessment (PG-SGA) for post-operative infections in patients with gastric cancer (GC) and colorectal cancer (CRC) who underwent curative surgery.

**Methods:**

This prospective study included 1,493 GC patients and 879 CRC patients who underwent curative surgery at 18 hospitals in China between April 2017 and March 2020. The NRS2002 and PG-SGA were performed on the day of admission. The relationship between the nutritional status of patients before surgery and post-surgical incidence of infection was analyzed using univariate and multiple logistic regression analyses.

**Results:**

According to NRS2002, the prevalence of nutritional risk was 51.1% in GC patients and 63.9% in CRC patients. According to the PG-SGA, 38.9% of GC patients and 54.2% of CRC patients had malnutrition. Approximately 4.4% of the GC patients and 9.9% of the CRC patients developed infectious complications after surgery. The univariate and multiple logistic regression analyses showed that the risk of infections was significantly higher in GC patients with a high nutritional risk score (NRS2002 ≥5) than in those with a low score (NRS2002 <3), and the PG-SGA score was identified as a predictor of post-operative infection complications of CRC.

**Conclusion:**

The pre-operative nutritional status of patients with GC or CRC has an impact on post-operative infection occurrence. NRS2002 ≥5 was a risk factor for post-operative infection in patients with GC, and the PG-SGA B/C was a predictor of infections in patients with CRC.

## Introduction

Colorectal cancer (CRC, including anal cancer) and gastric cancer (GC) are within the top five cancer types of all estimated cancer cases and deaths worldwide. CRC and GC represent the two major types of gastrointestinal cancers, accounting for 37.8 and 21.0% of the incidence, respectively (1). As common types of gastrointestinal tumors, CRC or GC in patients often gives rise to nutritional risk or malnutrition, which is exacerbated by surgical stress (2). In some patients with GC, skeletal muscle strength and mass decrease before surgery, which causes a vicious cycle of decline in physical function and further malnutrition, resulting in shortened survival (3, 4). Pre-operative nutrition and frailty have been reported to increase the relative risk of post-operative complications by 2–4 times (5, 6).

Infectious complications are one of the most common complications after surgery and are associated with poor prognosis. Infections after surgery can significantly increase hospitalization costs, prolong the length of hospital stay, and even lead to an increase in infection-related mortality (7). Therefore, the evaluation of perioperative risk factors is of great significance for the prevention and treatment of post-operative infections. In addition to age, BMI, ASA score, diabetes, multiple underlying diseases, and other factors (8, 9), nutritional risk and malnutrition are important risk factors for infections.

To increase awareness and allow for early recognition and treatment, many types of nutritional assessments are used in clinical practice, especially *via* validated nutrition screening tools. For example, the NRS2002 introduced by Kondrup et al., (10) is the preferred tool for screening and assessing hospital patients. The NRS2002 was developed by the Danish Society for Parenteral and Enteral Nutrition in 2003 and was verified in an analysis that included 128 controlled clinical trials (10). It was recommended to screen nutritional risk by the Europe Society for Parenteral and Enteral Nutrition (ESPEN) Guidelines (11). The patient-generated subjective global assessment (PG-SGA) tool, mentioned in the European guidelines, was modified according to the SGA and is a frequently used nutritional assessment tool in cancer patients (11).

However, their role in predicting post-operative infections in patients with gastrointestinal cancer is unknown. It has been shown that nutritional risk and low pre-operative nutritional status in patients with GC are associated with decreased immune function and the development of complications, especially infectious complications (12). However, Pacelli et al. (13) found that pre-operative nutritional status was not correlated with the incidence and mortality of post-operative infection-related complications in patients with GC. Hsueh et al. (14) compared five nutrition assessment tools and found that the PG-SGA performed the poorest and failed to predict any post-operative complications in patients with GC.

Previous studies generally included a range of diseases with a small number of cases, which may have led to inconsistent results. To date, there have been no multi-center studies in China on the relationship between NRS2002, PG-SGA, and post-operative infections in patients with gastrointestinal cancer. Thus, the aim of this study was to assess the prognostic value of NRS2002 and PG-SGA for post-operative infections in patients with GC and CRC who underwent curative surgery.

## Methods

This prospective cohort study was conducted in 18 hospitals in China between April 2017 and March 2020. The research protocol was reviewed and approved by the Ethics Committees of each institution. The National Ethics approval number is 2014ZFYJ-010. All the participants provided written informed consent. The Clinical Trial.gov identification number is NCT03115931.

### Patients

The main inclusion criteria were aged 18–80 years, diagnosed with GC or CRC, and scheduled to undergo elective surgical treatment. The main exclusion criteria included the presence of non-cancer inflammatory diseases, a history of malignant tumors, without curative surgery, an inability to complete the NRS2002 or PG-SGA, and a refusal to sign the consent form.

### Data Collection

The demographic and clinical characteristics were recorded within an electronic database by one or more trained investigators at each center. The weight and height were measured by two trained evaluators on the day of admission, and the body mass index (BMI) was calculated and classified according to the World Health Organization criteria. The diagnosed comorbidities (hypertension, diabetes), nutritional risk as determined by the NRS2002 at hospital admission, and smoking status (active smoker) were recorded. The PG-SGA was conducted on the day of admission. The Biochemical indexes, such as albumin, prealbumin, fasting plasma glucose, triglycerides, alanine aminotransferase, aspartate aminotransferase, total bilirubin, blood urea nitrogen (BUN), serum creatinine, hemoglobin, and white blood cell (WBC), were determined on the day of admission to the hospitals. In the present study, we examined infection complications classified according to the definition raised by the Centers for Disease Control and Prevention (15). This study particularly monitored the infections by the two experienced clinicians in each center. All infections were recorded between post-operative day 1 and hospital discharge.

#### NRS2002

Nutritional risk was assessed by NRS2002. NRS2002 takes into account impaired nutritional status (low, moderate or severe) and severity of disease (low, moderate or severe), with an adjustment for age of ≥70 years (10). The final scoring of NRS2002 ranges from 0 to 7. We use the three categories for the NRS2002: no nutritional risk (<3), nutritional risk (3, 4), and high nutritional risk (≥5). The NRS2002 was routinely conducted at admission by routine in the hospitals and recorded in the electronic medical record system. It was assessed by one trained nurse with 1-year nutritional expertise in each center.

#### PG-SGA

The PG-SGA, a nutritional status assessment method, was modified according to the SGA and designed specifically for cancer patients. This involves patients' self-assessment and medical staff evaluation. The core content includes seven parts: weight, food intake, symptoms, functional capacity, disease and its relation to nutritional requirements, metabolic demand (stress), and physical examination; the first four parts were evaluated by patients themselves and the last three by the medical staff (16). The examination consists of visual inspection and palpation of muscles, subcutaneous fat and edema. Muscle wasting was investigated by visual inspection and palpation of muscles with loss of bulk and tone in temporal areas, deltoids and quadriceps indicating muscle depletion. The triceps and midaxillary line at the level of the lower ribs were investigated with regard to depletion of subcutaneous fat. Ankles were examined for the presence of edema. The degree of muscle and fat depletion was evaluated and rated as 0 (normal) to 3 (severe deficit) (17). Based on the above assessments, patients were classified as well-nourished (PGSGA A), moderately malnourished (PG-SGA B) or severely malnourished (PG-SGA C).

The PG-SGA was carried out by trained registered clinical dietitians in each center, and supervised by one of our researchers. All dietitians underwent training in the PG-SGA procedure, as training has been shown to increase comprehensibility (18). We have provided a lecture explaining the rationale behind the PG-SGA, another lecture demonstrating its use and electronic version. Next, all the dietitians took part in a workshop to practice the PG-SGA, including the physical examination, and discussed the use and interpretation of the PG-SGA.

### Statistical Analysis

The continuous variables were described as means (standard deviations, SD) and the categorical variables as numbers (percentages). We preformed Pearson Chi-square test, Fisher's exact test and Cochran-Mantel-Haenszel Statistics on comparison of post-operative infection rates between gastric cancer and colorectal cancer and among different nutritional status. The odds ratios (ORs), 95% confidence intervals (95% CIs), and *P* for trend of the risk of post-operative infection complications were determined using univariate logistic regression models according to the three categories of the NRS2002 and PG-SGA and the quartiles of the serum biomarkers. Furthermore, the same method was used to calculate the ORs and 95% CIs for the serum biomarkers after log transformation and then dividing by the log transformation SD (per 1-SD increment). For the NRS2002 and PG-SGA, we developed two sets of multivariable-adjusted models: Model 1 adjusted for age and sex and Model 2 adjusted for age, gender, and other possible confounders that were identified in the univariate logistic regression analysis (*P* for trend <0.05). In addition, we performed subgroup analysis stratified by median age and sex for the risk of post-operative infection complications within the NRS2002 and PG-SGA groups. The dataset contains some missing values of the cancer stage and pre-operative chemotherapy. A sensitivity analysis was performed among those who had available information to increase the effect of the statistical analysis and interpret the main analysis. GC and CRC were to be analyzed separately in univariate and multivariable logistic regression and subgroup analysis. Statistical significance was set at *P* < 0.05. All the statistical analyses were performed using the SPSS version 25.0.

## Results

**Figure 1:**
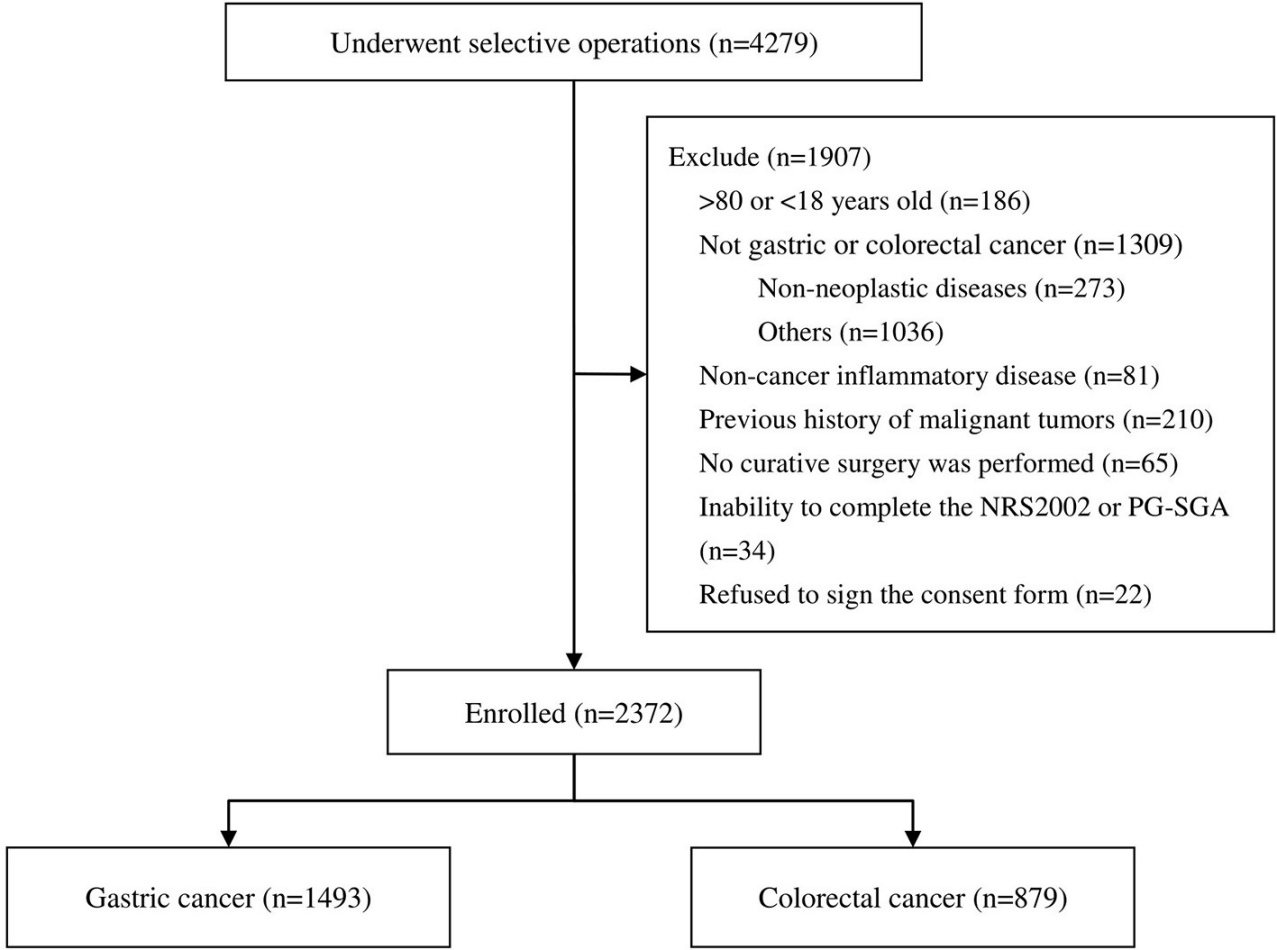
Diagram of patient enrollment in the study.

A total of 4,279 patients who underwent selective operations in general surgery departments between April 2017 and March 2020 were included. A total of 1,493 GC patients and 879 CRC patients fulfilled the criteria for enrollment in this study (Figure 1). Patients (*n* = 81) who had pre-operative non-cancer-related infectious diseases, those who did not undergo curative surgery (*n* = 65), patients who refused to sign consent form (*n* = 22), and patients with other conditions (*n* = 1,739) were excluded (Figure 1). Table 1 shows the characteristics of the study population. According to the NRS2002, in GC cohort, there were 48.9% had no nutritional risk, 45.1% had nutritional risk, and 6.0% had high nutritional risk. In CRC cohort, the proportions were 36.1, 56.9, and 7.1%. According to the PG-SGA, the percentages of patients who were well-nourished, moderately malnourished and severely malnourished in GC cohort were 61.1, 34.2, and 4.7%, respectively. And in CRC cohort were 45.8, 51.3, and 2.9%. The mean age of the GC patients was 59.8 ± 10.7 years, 72.5% were male, 2.0% had hypertension, and 8.7% had diabetes. Of the CRC patients, 60.8% were male and 39.2% female, with an average age of 60.2 ± 11.4 years. A total of 2.7% had a history of hypertension and 7.7% diabetes.

**Table 1:** Characteristics of all participants.

	**GC (*n* = 1,493)**	**CRC (*n* = 879)**	**Total (*n* = 2,372)**
Age, year	59.8 (10.7)	60.2 (11.4)	59.9 (11.0)
Gender
Male	1,082 (72.5)	534 (60.8)	1,616 (68.1)
Female	411 (27.5)	345 (39.2)	756 (31.9)
Height, cm	166.6 (7.3)	164.1 (8.1)	165.7 (7.7)
Weight, kg	64.5 (10.9)	62.8 (10.9)	63.8 (10.9)
BMI, kg/m^2^	23.2 (3.2)	23.2 (3.2)	23.2 (3.2)
Hypertension, yes	30 (2.0)	24 (2.7)	54 (2.3)
Diabetes, yes	130 (8.7)	68 (7.7)	198 (8.3)
Smoking, yes	390 (26.1)	172 (19.6)	562 (23.7)
NRS2002
<3	730 (48.9)	317 (36.1)	1,047 (44.1)
3–4	674 (45.1)	500 (56.9)	1,174 (49.5)
≥5	89 (6.0)	62 (7.1)	151 (6.4)
PG-SGA
A	895 (61.1)	400 (45.8)	1,295 (55.4)
B	501 (34.2)	448 (51.3)	949 (40.6)
C	69 (4.7)	26 (2.9)	95 (4.1)
Operation type
Laparotomy	1,068 (71.5)	361 (41.1)	1,429 (60.2)
Laparoscopy	425 (28.5)	518 (58.9)	943 (39.8)
Infection complications, yes	65 (4.4)	87 (9.9)	152 (6.4)
LOS, day	18.2 (8.6)	18.1 (7.9)	18.1 (8.3)
Albumin, g/L	41.5 (5.1)	40.6 (5.2)	41.2 (5.2)
Prealbumin, mg/L	266.0 (81.4)	260.5 (78.6)	263.9 (80.4)
FBG, mmol/L	5.6 (1.3)	5.6 (1.4)	5.6 (1.4)
TG, mmol/L	1.4 (0.8)	1.3 (0.7)	1.4 (0.8)
ALT, U/L	23.3 (20.6)	18.4 (12.4)	21.5 (18.2)
AST, U/L	26.7 (36.1)	20.9 (14.0)	24.5 (30.0)
T-Bil, μmol/L	13.2 (8.7)	13.6 (11.3)	13.3 (9.8)
BUN, mmol/L	5.7 (2.0)	5.2 (2.0)	5.5 (2.0)
Scr, μmol/L	75.4 (21.6)	73.1 (20.9)	74.6 (21.4)
Hemoglobin, g/L	127.4 (23.8)	126.2 (22.8)	126.9 (23.4)
WBC, 10^9^/L	5.9 (2.1)	6.7 (2.6)	6.2 (2.3)

*Quantitative variables were expressed as means (standard deviations). Categorical variables were expressed as number (percentages)*.

*GC, gastric cancer; CRC, colorectal cancer; BMI, body mass index; NRS2002, Nutrition Risk Screening 2002; PG-SGA, Patient-generated Subjective Global Assessment; LOS, length of stay; FBG, fasting plasma glucose; TG, triglycerides; ALT, alanine aminotransferase; AST, aspartate aminotransferase; T-Bil, total bilirubin; BUN, blood urea nitrogen; Scr, serum creatinine; Hb, hemoglobin; WBC, white blood cell*.

The number of post-operative infections per each NRS2002 and PG-SGA category was showed in Supplementary Table S1. We observed a gradual increase in the infection rate of CRC patients with the increase of NRS2002 scores, but it was not statistically significant (8.5, 10.4, and 12.9%, *P* = 0.485; Supplementary Table S1). A similar result was found in PG-SGA categories in GC patients (4.4, 3.8, and 8.7%, *P* = 0.192; Supplementary Table S1). The rates of post-operative infections between GC and CRC were statistically different [65 (4.4%) vs. 87 (9.9%) *P* < 0.001, Supplementary Table S1]. And the Test of Homogeneity of Odds Ratio with the NRS2002 categories suggested that the OR values between layers were homogenous (*P* = 0.456). Therefore, after controlling the NRS2002 influence on stratification factor, CRC was found to be a risk factor for the occurrences of infections, with a common-OR = 2.35, 95% CI 1.68–3.30, *P* < 0.001. However, the tests with PG-SGA categories suggested that the OR values between layers were heterogeneous (*P* = 0.047). Therefore, after PG-SGA stratification, CRC was found to be a risk factor for the occurrences of infections in patients with a “B” grade, OR = 3.70, 95% CI 2.16–6.32, *P* < 0.001. Those with an “A” or “C” grade, diagnosis had no effect on the occurrences of infections [OR (A) = 1.46, 95% CI 0.87–2.45, *P* (A) = 0.147; OR (C) = 2.50, 95% CI 0.69–9.04, *P* (C) = 0.166; Supplementary Table S1].

### Univariate Logistic Regression Analysis

In patients with GC, the univariate logistic regression analysis showed that smoking (OR 2.09, 95% CI: 1.26–3.46, *P* = 0.005; Table 2) was a significant risk factor for post-operative infections, whereas the NRS2002 scores were not (*P* for trend = 0.062; Table 3A). However, we found a significantly increased risk of post-operative infections in patients with NRS2002 ≥5 compared with those with NRS2002 <3 (OR 2.82, 95% CI: 1.29–6.19). For a per 1-SD increment in total bilirubin, the OR for infection post-operatively was 1.28 (95% CI: 1.01–1.62) and the BUN was 1.46 (95% CI: 1.15–1.86; Table 2).

**Table 2:** The association of clinical characteristics and hematologic biomarkers with post-operative infections.

		**GC**				**CRC**			
		**No. of cases**	**OR (95% CI)**	***P*-Value**	**Per 1-SD**	**No. of cases**	**OR (95% CI)**	***P*-Value**	**Per 1-SD**
Age		1,493	1.01 (0.99–1.04)	0.327		879	1.00 (0.99–1.02)	0.679	
Gender		1,493	0.71 (0.39–1.30)	0.271		879	0.80 (0.50–1.27)	0.338	
Smoking		1,493	2.09 (1.26–3.46)	0.005		879	1.26 (0.74–2.14)	0.398	
BMI		1,493	1.02 (0.95–1.10)	0.585		879	0.98 (0.92–1.06)	0.637	
Hypertension		1,493	0	0.998		879	3.99 (1.61–9.91)	0.003	
Diabetes		1,493	0.87 (0.34–2.20)	0.767		879	2.62 (1.39–4.95)	0.003	
Laparoscopy		1,493	2.39 (1.45–3.95)	0.001		879	0.23 (0.14–0.38)	<0.001	
Albumin	Q1	379	1	0.609^†^	0.94 (0.74–1.20)	220	1	0.630^†^	0.84 (0.68–1.03)
	Q2	382	0.99 (0.49–2.01)			236	0.80 (0.43–1.48)		
	Q3	361	1.54 (0.80–2.97)			204	1.04 (0.57–1.90)		
	Q4	371	0.63 (0.28–1.40)			219	0.78 (0.41–1.46)		
Prealbumin	Q1	380	1	0.188^†^	0.84 (0.66–1.07)	220	1	0.161^†^	0.87 (0.71–1.08)
	Q2	375	0.96 (0.50–1.86)			221	0.95 (0.53–1.71)		
	Q3	369	0.92 (0.47–1.79)			223	0.62 (0.32–1.17)		
	Q4	369	0.58 (0.27–1.24)			215	0.72 (0.39–1.35)		
FBG	Q1	376	1	0.479^†^	1.17 (0.93–1.48)	220	1	0.092^†^	1.15 (0.93–1.42)
	Q2	399	1.69 (0.82–3.48)			235	1.16 (0.60–2.23)		
	Q3	353	1.35 (0.62–2.92)			210	1.05 (0.53–2.08)		
	Q4	365	1.48 (0.70–3.15)			214	1.76 (0.95–3.27)		
TG	Q1	378	1	0.933^†^	1.05 (0.82–1.34)	228	1	0.838^†^	1.08 (0.86–1.35)
	Q2	392	0.90 (0.45–1.82)			214	1.07 (0.58–1.98)		
	Q3	350	0.95 (0.47–1.93)			222	0.65 (0.33–1.27)		
	Q4	373	1.01 (0.51–2.02)			215	1.23 (0.68–2.22)		
ALT	Q1	385	1	0.819^†^	1.08 (0.84–1.37)	221	1	0.096^†^	1.21 (0.97–1.50)
	Q2	407	1.51 (0.76–3.00)			227	1.83 (0.93–3.62)		
	Q3	351	1.02 (0.47–2.20)			212	1.80 (0.90–3.60)		
	Q4	350	1.27 (0.61–2.64)			219	1.91 (0.96–3.77)		
AST	Q1	419	1	0.911^†^	1.06 (0.83–1.34)	232	1	0.083^†^	1.17 (0.95–1.43)
	Q2	379	1.25 (0.64–2.44)			229	1.58 (0.82–3.06)		
	Q3	328	0.90 (0.42–1.91)			212	1.56 (0.80–3.06)		
	Q4	367	1.15 (0.58–2.28)			206	1.87 (0.97–3.60)		
T-Bil	Q1	387	1	0.078^†^	1.28 (1.01–1.62)	220	1	0.059^†^	1.15 (0.92–1.42)
	Q2	365	1.62 (0.72–3.64)			221	0.72 (0.35–1.47)		
	Q3	375	2.46 (1.16–5.25)			219	1.49 (0.80–2.76)		
	Q4	366	1.84 (0.83–4.07)			219	1.49 (0.80–2.76)		
BUN	Q1	359	1	0.089^†^	1.46 (1.15–1.86)	206	1	0.818^†^	1.06 (0.84–1.35)
	Q2	358	0.52 (0.23–1.18)			205	1.13 (0.58–2.20)		
	Q3	356	0.70 (0.33–1.49)			202	1.02 (0.52–2.03)		
	Q4	341	1.59 (0.84–3.00)			204	0.95 (0.48–1.9)		
Scr	Q1	369	1	0.756^†^	0.87 (0.72–1.05)	224	1	0.186^†^	0.87 (0.70–1.07)
	Q2	368	1.00 (0.49–2.04)			205	0.96 (0.52–1.76)		
	Q3	373	1.18 (0.60–2.34)			215	0.86 (0.47–1.59)		
	Q4	363	0.82 (0.39–1.73)			214	0.64 (0.33–1.24)		
Hb	Q1	378	1	0.427^†^	1.17 (0.89–1.54)	221	1	0.970^†^	0.97 (0.78–1.21)
	Q2	390	1.26 (0.62–2.57)			227	1.07 (0.58–1.97)		
	Q3	360	1.05 (0.49–2.24)			223	0.84 (0.44–1.60)		
	Q4	365	1.43 (0.71–2.89)			208	1.07 (0.57–2.00)		
WBC	Q1	376	1	0.496^†^	0.97 (0.75–1.24)	221	1	0.058^†^	1.25 (1.01–1.55)
	Q2	376	0.73 (0.36–1.47)			219	1.01 (0.52–1.96)		
	Q3	371	1.01 (0.53–1.95)			221	0.89 (0.45–1.75)		
	Q4	370	0.68 (0.33–1.41)			218	1.83 (1.00–3.34)		

*GC, gastric cancer; CRC, colorectal cancer; FBG, fasting plasma glucose; TG, triglycerides; ALT, alanine aminotransferase; AST, aspartate aminotransferase; T-Bil, total bilirubin; BUN, blood urea nitrogen; Scr, serum creatinine; Hb, hemoglobin; WBC, white blood cell*.

† *For trend*.

**Table 3A:** The association of NRS2002 and PG-SGA with post-operative infections (GC).

		**OR (95% CI)**			***P* for trend**	**Per 1-SD**
		**Category 1**	**Category 2**	**Category 3**		
NRS2002^†^	**No. of cases**	730	674	89		
	Not adjusted	1	1.09 (0.64–1.86)	2.82 (1.29–6.19)	0.062	1.27 (1.00–1.61)
	**No. of cases**	730	674	89		
	Model 1	1	1.09 (0.63–1.86)	2.75 (1.22–6.19)	0.078	1.26 (0.99–1.60)
	**No. of cases**	696	639	79		
	Model 2	1	0.99 (0.57–1.73)	1.95 (0.81–4.69)	0.325	1.17 (0.91–1.51)
PG-SGA^‡^	**No. of cases**	895	501	69		
	Not adjusted	1	0.87 (0.49–1.51)	2.09 (0.85–5.13)	0.499	1.09 (0.85–1.38)
	**No. of cases**	895	501	69		
	Model 1	1	0.87 (0.49–1.52)	2.10 (0.85–5.17)	0.499	1.09 (0.85–1.39)
	**No. of cases**	840	479	69		
	Model 2	1	0.80 (0.45–1.45)	1.86 (0.73–4.77)	0.705	1.05 (0.82–1.35)

*Model 1: Adjusted for age and gender. Model 2: Adjusted for age, gender, smoking, hypertension, diabetes, laparoscopy, T-Bil, BUN and WBC. GC, gastric cancer*.

† *NRS2002 was classified as three categories by the criteria of “ <3,” “3–4,” and “≥5” score. PG-SGA was classified as three categories by the criteria of “A,” “B,” and “C” grade*.

From the univariate logistic regression analysis of the CRC patients, hypertension (OR 3.99, 95% CI: 1.61–9.91, *P* = 0.003; Table 2), diabetes (OR 2.62, 95% CI: 1.39–4.95, *P* = 0.003; Table 2), and the PG-SGA B/C (*P* for trend <0.001; Table 3B) were predictors of post-operative infection complications, while laparoscopic surgery (OR 0.23, 95% CI: 0.14–0.38, *P* < 0.001; Table 2) was a protective factor. In patients with CRC, NRS2002 score <3 category was set as the reference, and the OR for the risk of post-operative infections for patients in the NRS2002 score = 3–4 category and the NRS2002 score ≥5 category was 1.25 (95% CI: 0.77–2.03) and 1.59 (95% CI: 0.69–3.69), respectively (*P* for trend = 0.232; Table 3B). With the increase of NRS2002 score, the upward trend of infection risk was not statistically significant. For a per 1-SD increment in WBC, the OR for post-operative infections was 1.25 (95% CI: 1.01–1.55; Table 2).

**Table 3B:** The association of NRS2002 and PG-SGA with post-operative infections (CRC).

		**OR (95% CI)**			***P* for trend**	**Per 1-SD**
	**Category 1**	**Category 2**	**Category 3**		
NRS2002^†^	**No. of cases**	317	500	62		
	Not adjusted	1	1.25 (0.77–2.03)	1.59 (0.69–3.69)	0.232	1.29 (1.03–1.61)
	**No. of cases**	317	500	62		
	Model 1	1	1.26 (0.77–2.05)	1.58 (0.67–3.75)	0.234	1.29 (1.03–1.62)
	**No. of cases**	303	456	58		
	Model 2	1	1.04 (0.60–1.81)	1.43 (0.57–3.60)	0.550	1.19 (0.93–1.53)
PG-SGA^‡^	**No. of cases**	400	448	26		
	Not adjusted	1	2.19 (1.34–3.57)	3.57 (1.24–10.27)	0.001	1.48 (1.19–1.85)
	**No. of cases**	400	448	26		
	Model 1	1	2.22 (1.36–3.63)	3.57 (1.23–10.33)	<0.001	1.49 (1.19–1.86)
	**No. of cases**	371	415	26		
	Model 2	1	2.62 (1.48–4.63)	5.29 (1.64–17.10)	<0.001	1.64 (1.27–2.12)

*Model 1: Adjusted for age and gender. Model 2: Adjusted for age, gender, smoking, hypertension, diabetes, laparoscopy, T-Bil, BUN, and WBC. CRC, colorectal cancer*.

† *NRS2002 was classified as three categories by the criteria of “ <3,” “3–4,” and “≥5” score. PG-SGA was classified as three categories by the criteria of “A,” “B,” and “C” grade*.

### Multivariable Logistic Regression Analysis

The results of multivariable logistic regression analysis are presented in Tables 3A,B. In Model 1 of the GC patients, the OR for post-operative infections was 2.75 (95% CI: 1.22–6.19; Table 3A) in individuals with NRS2002 ≥5 compared with those with NRS2002 <3. NRS2002 ≥5 was not associated with post-operative infection complications in Model 2 (OR 1.95, 95% CI: 0.81–4.69; Table 3A). In both of the models, the PG-SGA B/C remained an important predictor of infection complications in the CRC patients compared with PG-SGA A (*P* for trend <0.001; Table 3B) but was not a significant predictor in the GC patients (*P* for trend >0.05; Table 3A). The results of sensitivity analysis were similar to the main analysis (Supplementary Tables S3A,B).

### Stratified Analysis

Based on stratified analysis, the OR of NRS2002 ≥5 was higher in the younger subgroup ( ≤ 61 years) of GC (OR 3.68, 95% CI: 1.15–11.72, *P* for interaction = 0.043; Table 4A) according to the median age of the patients. In the male population with GC, the OR of NRS2002 ≥5 was statistically significant (OR 3.09, 95% CI: 1.26–7.59, *P* for trend = 0.045; Table 4A). In the stratified analysis of patients with CRC patients, the NRS2002 score remained statistically insignificant; meanwhile, PG-SGA grade was not correlated with post-operative infections in the younger subgroup ( ≤ 62 years; *P* for trend = 0.144, *P* for interaction = 0.043; Table 4B). However, in the older subgroup, the OR of each category of PG-SGA increased (PG-SGA B vs. A: OR 3.23, 95% CI:1.44–7.25; PG-SGA C vs. A: OR 6.79, 95% CI:1.79–25.83; *P* for trend = 0.001, *P* for interaction <0.001; Table 4B).

**Table 4A:** Stratified analysis of GC (age and gender).

			**OR (95% CI)**			***P* for trend**	**Per 1-SD**	***P* for interaction**
			**Category 1**	**Category 2**	**Category 3**			
NRS2002^†^	Age	**No. of cases**	415	328	28			
		≤ 61^*^	1	0.55 (0.24–1.29)	3.68 (1.15–11.72)	0.729	1.19 (0.83–1.71)	0.043
		**No. of cases**	315	346	61			
		>61^*^	1	1.87 (0.86–4.06)	2.72 (0.90–8.27)	0.046	1.31 (0.95–1.80)	
	Gender	**No. of cases**	540	479	63			
		Male	1	1.25 (0.68–2.28)	3.09 (1.26–7.59)	0.045	1.31 (1.00–1.71)	0.706
		**No. of cases**	190	195	26			
		Female	1	0.69 (0.21–2.21)	2.18 (0.43–11.10)	0.792	1.16 (0.70–1.93)	
PG-SGA^‡^	Age	**No. of cases**	467	257	33			
		≤ 61^*^	1	0.80 (0.34–1.87)	3.44 (1.09–10.83)	0.303	1.20 (0.85–1.69)	0.402
		**No. of cases**	428	244	36			
		>61^*^	1	0.92 (0.43–1.93)	1.14 (0.26–5.07)	0.958	0.99 (0.70–1.39)	
	Gender	**No. of cases**	656	354	46			
		Male	1	1.16 (0.63–2.12)	1.51 (0.44–5.15)	0.468	1.11 (0.84–1.46)	0.788
		**No. of cases**	239	147	23			
		Female	1	0.16 (0.02–1.24)	3.44 (0.87–13.50)	0.859	1.05 (0.63–1.74)	

*GC, gastric cancer*.

† *NRS2002 was classified as three categories by the criteria of “ <3,” “3–4,” and “≥5” score. PG-SGA was classified as three categories by the criteria of “A,” “B,” and “C” grade*.

* *The median age of the GC patients is 61*.

**Table 4B:** Stratified analysis of CRC (age and gender).

			**OR (95% CI)**			***P* for trend**	**Per 1-SD**	***P* for interaction**
			**Category 1**	**Category 2**	**Category 3**			
NRS2002^†^	Age	**No. of cases**	173	283	14			
		≤ 62^*^	1	1.16 (0.60–2.23)	1.76 (0.36–8.59)	0.513	1.20 (0.85–1.69)	0.238
		**No. of cases**	144	217	48			
		>62^*^	1	1.37 (0.66–2.83)	1.57 (0.56–4.44)	0.330	1.35 (1.00–1.82)	
	Gender	**No. of cases**	200	300	34			
		Male	1	1.08 (0.60–1.94)	1.55 (0.54–4.46)	0.515	1.22 (0.93–1.62)	0.802
		**No. of cases**	117	200	28			
		Female	1	1.75 (0.72–4.26)	1.89 (0.46–7.81)	0.231	1.44 (0.98–2.11)	
PG-SGA^‡^	Age	**No. of cases**	229	229	10			
		≤ 62^*^	1	1.67 (0.88–3.15)	1.39 (0.17–11.59)	0.144	1.26 (0.92–1.72)	<0.001
		**No. of cases**	171	219	16			
		>62^*^	1	3.23 (1.44–7.25)	6.79 (1.79–25.83)	0.001	1.76 (1.27–2.44)	
	Gender	**No. of cases**	254	259	16			
		Male	1	1.87 (1.04–3.37)	4.12 (1.21–14.03)	0.008	1.44 (1.10–1.89)	0.094
		**No. of cases**	146	189	10			
		Female	1	3.23 (1.28–8.16)	2.59 (0.28–23.91)	0.019	1.6 (1.08–2.37)	

*CRC, colorectal cancer*.

† *NRS2002 was classified as three categories by the criteria of “ <3,” “3–4,” and “≥5” score. PG-SGA was classified as three categories by the criteria of “A,” “B,” and “C” grade*.

* *The median age of the CRC patients is 62*.

## Discussions

Gastric cancer and CRC had the 5th (5.6%) and 3rd (10%) highest incidences among all cancers, respectively, and the 4th (7.7%) and 2nd (9.4%) for mortality, respectively, according to Global Cancer Statistics 2020 (1). Pre-operative nutritional status is associated with short-term and long-term prognosis in gastrointestinal cancer (19–21). The aim of this study was to assess the relationship between the pre-operation nutritional status and post-operation infections in patients with GC and CRC. In our study, 51.1% of the GC patients and 63.9% of the CRC patients were at nutritional risk according to the NRS2002, and 38.9 and 54.2% had moderate or severe malnutrition, respectively, according to the PG-SGA. We found that the PG-SGA B and C was a risk factor for post-operative infections in CRC. In patients with GC, NRS2002 ≥5 was a risk factor.

There are several commonly used nutritional screening tools worldwide, including the NRS2002, malnutrition screening tools (MST), and malnutrition universal screening tools (MUST). The most widely used is NRS2002 because of its low cost, easy application, and wide applicability. In contrast to the previous nutrition score, it assessed the severity of the disease to evaluate nutritional requirements. In addition, age was also taken into account. All of these features enabled the NRS2002 tool to cover a wide range of diseases in hospital, including cancer.

We found that having a high nutritional risk, defined as NRS2002 ≥5, increased the risk of post-operative infections in patients with GC, which was similar to what was reported in previous studies. Qiu et al. (22) reported that having NRS2002 ≥3 was an independent adverse prognostic factor for overall survival in their study that included 830 patients with GC. In another study that included 880 GC patients who were undergoing a gastrectomy, NRS2002 ≥3 was significantly associated with post-operative complications (*P* < 0.001). However, this association disappeared when the authors performed multivariate analyses adjusting for sex, age, BMI, Charlson comorbidity index, hypohemia, hypoprotein malnutrition, tumor site, laparoscopic surgery, and sarcopenia (23). However, in our study, NRS2002 ≥5 maintained consistent prediction ability in the multifactor-adjusted models. Therefore, we believe that NRS2002 ≥5 is more predictable than NRS2002 ≥3 in indicating poor prognosis of the patient underwent radical gastrectomy. In addition, in stratified analysis of NRS2002 ≥5, we observed that the risk of post-operative infections was higher in GC patients younger than 61 years and in male GC patients, and it was lower in the patients older than 61 years and in female. However, the results in the age stratification were not statistically significant. Nutritional risk evaluated by the NRS2002 was not identified as a predictor of post-operative infections in patients with CRC. The results of Wang et al. reflected the same view. They found that there were no statistically significant differences in the incidence rates of post-operative complications between patients with and without nutritional risk, according to NRS2002 score (*P* = 0.546) (24). In contrast to the present findings, a previous study performed by Schwegler et al. (25) suggested that the NRS2002 was successful in predicting post-operative complications, including infections, in 186 CRC patients who were undergoing surgery (OR 2.43, *P* = 0.004). Correspondingly, our study was a multi-center study with a large sample size, and our outcome focused on infection complications, which was the advantage of our study compared with the former.

The NRS2002 is a screening tool, which is a fast and simple method that can be used by any healthcare professional to determine whether patients need further comprehensive nutritional assessment and nutritional treatment plan. In the meanwhile, guidelines suggest that objective and quantitative assessments should be applied to patients with abnormal nutritional screening results (11). There are several commonly used nutrition assessment tools, including the PG-SGA, Mini-Nutritional Assessment (MNA), and Global Leadership Initiative on Malnutrition (GLIM). Due to its comprehensiveness and utility, the PG-SGA appears to be one of the most useful methods for the nutritional assessment of cancer patients. As an assessment tool, the PG-SGA should be conducted by nutrition professionals, which includes a comprehensive examination and evaluation of the patients' nutrition metabolism and body function for establishing a nutritional treatment plan.

We observed that the risk of post-operative infections increased in patients with CRC in the PG-SGA B group 2.19 times (95% CI: 1.34–3.57) and the PG-SGA C group 3.57 times (95% CI: 1.24–10.27). The prognostic value of the PG-SGA for post-operative infections maybe attributed to the combination of data such as unconscious weight loss, food intake, gastrointestinal symptoms, active capability, and physical examination of the patient, which were strongly associated with negative outcomes (26, 27). In multivariable logistic regression analysis, the ORs of PG-SGA B/C increased (PG-SGA B vs. A: OR 2.62, 95% CI: 1.48–4.63; PG-SGA C vs. A: OR 5.29, 95% CI: 1.64–17.10; *P* for trend <0.001), which means that the prognostic value of the PG-SGA for post-operative infections increased after adjusting for age, gender, smoking, hypertension, diabetes, operation type, total bilirubin, BUN, and WBC. However, when we stratified age, we found that PG-SGA lost its predictive power for people younger than 62 years. This leads us to believe that PG-SGA may be more suitable for predicting post-operative infections in elderly colorectal cancer patients. Our study also suggested that the PG-SGA was unable to predict post-operative infections in patients with GC. Similar to this study, Seo et al. (20) found that the PG-SGA did not predict the adverse events of post-operative chemotherapy in patients with gastrectomy. Esfahani et al. (28) found that there was no significant difference in the PG-SGA scores between metastatic and non-metastatic GC patients. The reason for the weakness of the PG-SGA in predicting poor prognosis of GC patients is unknown. Meanwhile, a more suitable nutritional status assessment tool for patients with GC is still needed.

This study has a few limitations that need to be considered. Although we performed nutritional screening and assessment during the first day of admission, there were no records of NRS2002 score and PG-SGA grade after that. Therefore, we cannot guarantee that the initial NRS2002 score and PG-SGA grade had been unaltered before surgery. Significant improvement in dietitians' perception of difficulty and comprehensibility of the PG-SGA can be achieved by providing short term training. However, a perceived difficulty for the physical examination still remained, which may have affected the classifying the degree of malnutrition. Another limitation is that we did not consider the impact of perioperative nutritional support therapy. In our 18 centers, the medical staff would provide nutritional support therapy for patients with nutritional risk or malnutrition in accordance with the local practices, and there is still no uniform standard. Therefore, we ignored the effects of nutrition support therapy on results in this study. In a subsequent study, we will continue to explore the relationship between nutritional support therapy and the prognosis of cancer.

In conclusion, this study showed that NRS2002 ≥5 provided good value for clinicians in the prediction of post-operative infections in patients with GC, enabling advanced interventions. On the other hand, PG-SGA B/C was a good predictor in CRC patients. Simultaneously, this article highlighted the need for a nutritional assessment tool that can better predict clinical outcomes in patients with GC.

## Data Availability Statement

The raw data supporting the conclusions of this article will be made available by the authors, without undue reservation.

## Ethics Statement

The studies involving human participants were reviewed and approved by Clinical Trial Ethics Committee, Jinling Hospital. The patients/participants provided their written informed consent to participate in this study.

## Author Contributions

XWa, LZ, and XG contributed to the conception and design of the study. LZ and SW drafted and revised the manuscript. XWa supervised the entire project and was responsible for the conception and funding. TG collected and collated data. DH contributed to the statistical analysis plan. SW contributed to the collation and analysis of the data. XWa and DH revised the manuscript for important intellectual content. LH, BL, YG, JC, DG, ZJ, YW, FG, JZ, ZX, ZC, JX, LW, JQ, GD, HH, YN, GaL, ML, HY, WZ, YZ, HQ, XWu, KW, QC, JY, YT, PZ, GJ, BO, and GuL contributed to case enrollment and data collection. All authors critically revised the manuscript, agreed to be fully accountable for ensuring the integrity and accuracy of the work, read, and approved the final manuscript. All authors contributed to the article and approved the submitted version.

## Funding

This study was supported by the Research Special Fund for Public Welfare Industry of Health (201502022) and the National Natural Science Foundation of China (81700518).

## Conflict of Interest

The authors declare that the research was conducted in the absence of any commercial or financial relationships that could be construed as a potential conflict of interest.

## Publisher's Note

All claims expressed in this article are solely those of the authors and do not necessarily represent those of their affiliated organizations, or those of the publisher, the editors and the reviewers. Any product that may be evaluated in this article, or claim that may be made by its manufacturer, is not guaranteed or endorsed by the publisher.
