# Supplementary material for: Poor Pre-operative Nutritional Status Is a Risk Factor of Post-operative Infections in Patients With Gastrointestinal Cancer—A Multicenter Prospective Cohort Study
Source: Front Nutr. 2022 May 27;9:850063. doi: 10.3389/fnut.2022.850063 (PMC9184816; doi:10.3389/fnut.2022.850063)
Supplement: Supplementary file 1 [file Data_Sheet_1.docx]

Supplementary Material

**Supplementary Table 1.** Chi–square test for types of diseases and nutritional status

|  | | GC | | CRC | | *P*-value ^‡^ | OR (95% CI) ^‡^ | Tests of Homogeneity of the Odds Ratio (*P*-value) ^‡^ |
| --- | --- | --- | --- | --- | --- | --- | --- | --- |
|  |  | Infection，n (%) | *P*-value | Infection，n (%) | *P*-value |  |  |  |
| NRS2002 | <3 | 28 (3.8%) | 0.038^§^ | 27 (8.5%) | 0.485 | 0.002 | 2.33 (1.35–4.03) | 0.456 |
|  | 3-4 | 28 (4.2%) |  | 52 (10.4%) |  | <0.001 | 2.68 (1.67–4.31) |  |
|  | ≥5 | 9 (10.1%) |  | 8 (12.9%) |  | 0.594 | 1.32 (0.48–3.63) |  |
| Total | | 65 (4.4%) |  | 87 (9.9%) |  | <0.001 | 2.35 (1.68–3.30) |  |
| PG-SGA | A | 39 (4.4%) | 0.192^†^ | 25 (6.3%) | 0.001^※^ | 0.147 | 1.46 (0.87–2.45) | 0.047 |
|  | B | 19 (3.8%) |  | 57 (12.7%) |  | <0.001 | 3.70 (2.16–6.32) |  |
|  | C | 6 (8.7%) |  | 5 (19.2%) |  | 0.166^†^ | 2.50 (0.69–9.04) |  |
| Total | | 65 (4.4%) |  | 87 (9.9%) |  | <0.001 | 2.39 (1.69–3.37) |  |

GC: gastric cancer, CRC: colorectal cancer.

†: Fisher's exact test. ‡: Cochran-Mantel-Haenszel Statistics. §: NRS2002 “<3” vs. “3–4” *P*=0.760, NRS2002 “<3” vs. “≥5” *P*=0.013, NRS2002 “3–4” vs. “≥5” *P*=0.030. ※: PG-SGA “A” vs. “B” *P*=0.001, PG-SGA “A” vs. “C” *P*=0.028, PG-SGA “B” vs. “C” *P*=0.365.

**Supplementary Table 2.** The association of clinical characteristics and hematologic biomarkers with postoperative infection (In the cohort which the stage of cancer and previous treatment was available)

|  |  | GC | | | | CRC | | | |
| --- | --- | --- | --- | --- | --- | --- | --- | --- | --- |
|  |  | No. of cases | OR (95% CI) | *P-value* | Per 1–SD | No. of cases | OR (95% CI) | *P-value* | Per 1–SD |
| Age |  | 1004 | 1.02 (0.99–1.05) | 0.217 |  | 457 | 1.02 (0.99–1.05) | 0.276 |  |
| Gender |  | 1004 | 0.83 (0.42–1.65) | 0.592 |  | 457 | 0.65 (0.33–1.27) | 0.205 |  |
| Smoking |  | 1004 | 1.70 (0.93–3.11) | 0.086 |  | 457 | 1.01 (0.47–2.17) | 0.988 |  |
| BMI |  | 1004 | 1.03 (0.95–1.13) | 0.466 |  | 457 | 0.96 (0.87–1.05) | 0.356 |  |
| Hypertension |  | 1004 | 0 | 0.998 |  | 457 | 4.13 (1.03–16.58) | 0.045 |  |
| [Diabetes](C:/Program%20Files%20(x86)/Youdao/Dict/8.9.9.0/resultui/html/index.html#/javascript:;) |  | 1004 | 0.86 (0.30–2.46) | 0.785 |  | 457 | 2.53 (1.13–5.67) | 0.024 |  |
| Laparoscopy |  | 1004 | 2.92 (1.62–5.27) | <0.001 |  | 457 | 0.32 (0.16–0.62) | 0.001 |  |
| [Albumin](#/javascript:;) | Q1 | 254 | 1 | 0.691^†^ | 0.91 (0.69–1.19) | 115 | 1 | 0.309^†^ | 0.74 (0.56–0.98) |
|  | Q2 | 255 | 1.28 (0.57–2.88) |  |  | 125 | 0.52 (0.22–1.23) |  |  |
|  | Q3 | 245 | 1.24 (0.54–2.82) |  |  | 103 | 0.96 (0.44–2.13) |  |  |
|  | Q4 | 250 | 0.83 (0.34–2.03) |  |  | 114 | 0.50 (0.20–1.24) |  |  |
| Prealbumin | Q1 | 265 | 1 | 0.053^†^ | 0.75 (0.57–0.99) | 115 | 1 | 0.002^†^ | 0.60 (0.46–0.79) |
|  | Q2 | 237 | 1.13 (0.54–2.36) |  |  | 123 | 0.32 (0.14–0.72) |  |  |
|  | Q3 | 253 | 0.76 (0.34–1.68) |  |  | 106 | 0.16 (0.05–0.47) |  |  |
|  | Q4 | 249 | 0.41 (0.16–1.08) |  |  | 113 | 0.35 (0.15–0.79) |  |  |
| FBG | Q1 | 291 | 1 | 0.639^†^ | 1.10 (0.83–1.46) | 115 | 1 | 0.764^†^ | 1.06 (0.78–1.43) |
|  | Q2 | 212 | 1.27 (0.55–2.94) |  |  | 118 | 1.41 (0.60–3.33) |  |  |
|  | Q3 | 254 | 1.05 (0.46–2.43) |  |  | 111 | 0.82 (0.31–2.15) |  |  |
|  | Q4 | 247 | 1.29 (0.58–2.89) |  |  | 113 | 1.37 (0.57–3.25) |  |  |
| TG | Q1 | 259 | 1 | 0.597^†^ | 0.92 (0.68–1.24) | 116 | 1 | 0.417^†^ | 0.92 (0.68–1.25) |
|  | Q2 | 245 | 1.06 (0.48–2.34) |  |  | 114 | 0.72 (0.32–1.64) |  |  |
|  | Q3 | 250 | 0.79 (0.34–1.83) |  |  | 117 | 0.43 (0.17–1.09) |  |  |
|  | Q4 | 250 | 0.87 (0.38–1.98) |  |  | 110 | 0.82 (0.37–1.85) |  |  |
| ALT | Q1 | 266 | 1 | 0.766^†^ | 0.92 (0.68–1.24) | 115 | 1 | 0.413^†^ | 1.16 (0.85–1.57) |
|  | Q2 | 236 | 1.69 (0.77–3.71) |  |  | 116 | 1.69 (0.67–4.24) |  |  |
|  | Q3 | 263 | 0.82 (0.34–2.02) |  |  | 112 | 1.61 (0.63–4.09) |  |  |
|  | Q4 | 239 | 1.12 (0.48–2.63) |  |  | 114 | 1.57 (0.62–4.01) |  |  |
| AST | Q1 | 291 | 1 | 0.651^†^ | 0.89 (0.65–1.23) | 121 | 1 | 0.411^†^ | 1.13 (0.85–1.51) |
|  | Q2 | 255 | 1.43 (0.68–3.04) |  |  | 115 | 1.96 (0.79–4.86) |  |  |
|  | Q3 | 209 | 0.74 (0.29–1.89) |  |  | 107 | 1.62 (0.63–4.19) |  |  |
|  | Q4 | 249 | 0.99 (0.44–2.25) |  |  | 114 | 1.66 (0.65–4.23) |  |  |
| T-Bil | Q1 | 257 | 1 | 0.107^†^ | 1.24 (0.94–1.65) | 115 | 1 | 0.700^†^ | 0.95 (0.70–1.30) |
|  | Q2 | 253 | 2.73 (0.96–7.77) |  |  | 115 | 0.43 (0.16–1.18) |  |  |
|  | Q3 | 244 | 4.01 (1.47–10.99) |  |  | 113 | 1.11 (0.50–2.48) |  |  |
|  | Q4 | 250 | 2.32 (0.79–6.78) |  |  | 114 | 0.92 (0.40–2.12) |  |  |
| BUN | Q1 | 259 | 1 | 0.005^†^ | 1.81 (1.39–2.38) | 117 | 1 | 0.915^†^ | 1.19 (0.87–1.61) |
|  | Q2 | 244 | 0.52 (0.18–1.55) |  |  | 114 | 1.03 (0.44–2.40) |  |  |
|  | Q3 | 257 | 1.01 (0.41–2.47) |  |  | 114 | 0.75 (0.30–1.86) |  |  |
|  | Q4 | 244 | 2.47 (1.14–5.32) |  |  | 112 | 1.05 (0.45–2.45) |  |  |
| Scr | Q1 | 258 | 1 | 0.682^†^ | 0.86 (0.69–1.07) | 115 | 1 | 0.202^†^ | 0.86 (0.65–1.13) |
|  | Q2 | 244 | 0.98 (0.44–2.18) |  |  | 114 | 0.64 (0.28–1.49) |  |  |
|  | Q3 | 253 | 0.86 (0.38–1.95) |  |  | 129 | 0.75 (0.34–1.65) |  |  |
|  | Q4 | 249 | 0.87 (0.38–1.98) |  |  | 99 | 0.51 (0.20–1.30) |  |  |
| Hb | Q1 | 253 | 1 | 0.868^†^ | 1.07 (0.79–1.45) | 122 | 1 | 0.820^†^ | 0.95 (0.70–1.28) |
|  | Q2 | 268 | 1.12 (0.49–2.55) |  |  | 107 | 0.62 (0.25–1.55) |  |  |
|  | Q3 | 248 | 1.12 (0.48–2.59) |  |  | 117 | 0.64 (0.27–1.55) |  |  |
|  | Q4 | 235 | 1.08 (0.46–2.54) |  |  | 111 | 1.11 (0.51–2.45) |  |  |
| WBC | Q1 | 251 | 1 | 0.350^†^ | 0.82 (0.61–1.11) | 115 | 1 | 0.178^†^ | 1.22 (0.90–1.64) |
|  | Q2 | 257 | 0.83 (0.38–1.83) |  |  | 114 | 1.24 (0.51–2.99) |  |  |
|  | Q3 | 250 | 0.85 (0.39–1.88) |  |  | 114 | 0.38 (0.12–1.26) |  |  |
|  | Q4 | 246 | 0.64 (0.27–1.51) |  |  | 114 | 2.10 (0.93–4.74) |  |  |

GC: gastric cancer, CRC: colorectal cancer, FBG: fasting plasma glucose, TG: triglycerides, ALT: alanine aminotransferase, AST: aspartate aminotransferase, T-Bil: total bilirubin, BUN: blood urea nitrogen, Scr: serum creatinine, Hb: hemoglobin, WBC: white blood cell.

†: For trend.

**Supplementary Table 3A.** The association of NRS2002 and PG-SGA with postoperative infection (In the cohort which the stage of cancer and previous treatment was available in GC)

|  | | OR (95%CI) | | | *P* for trend | Per 1–SD |
| --- | --- | --- | --- | --- | --- | --- |
|  |  | Category 1 | Category 2 | Category 3 |  |  |
| NRS2002^†^ | No. of cases | 510 | 447 | 47 |  |  |
|  | Not adjusted | 1 | 0.99 (0.53–1.84) | 3.25 (1.25–8.46) | 0.149 | 1.22 (0.93–1.62) |
|  | No. of cases | 510 | 447 | 47 |  |  |
|  | Model 1 | 1 | 0.96 (0.51–1.81) | 2.94 (1.09–7.93) | 0.219 | 1.19 (0.90–1.58) |
|  | No. of cases | 510 | 447 | 47 |  |  |
|  | Model 2 | 1 | 0.88 (0.46–1.72) | 1.97 (0.68–5.68) | 0.528 | 1.11 (0.83–1.49) |
| PG-SGA^‡^ | No. of cases | 641 | 317 | 46 |  |  |
|  | Not adjusted | 1 | 0.98 (0.51–1.87) | 2.01 (0.68–5.99) | 0.461 | 1.11 (0.84–1.47) |
|  | No. of cases | 641 | 317 | 46 |  |  |
|  | Model 1 | 1 | 0.96 (0.50–1.84) | 1.90 (0.63–5.68) | 0.522 | 1.10 (0.83–1.45) |
|  | No. of cases | 641 | 317 | 46 |  |  |
|  | Model 2 | 1 | 1.17 (0.57–2.43) | 0.79 (0.19–3.28) | 0.979 | 1.11 (0.78–1.57) |

Model 1: Adjusted for age and gender. Model 2: Adjusted for age, gender, hypertension, diabetes, laparoscopy, albumin, prealbumin and BUN. GC: gastric cancer.

†: NRS2002 was classified as three categories by the criteria of “<3”, “3–4” and “≥5” score. ‡: PG-SGA was classified as three categories by the criteria of “A”, “B” and “C” grade.

**Supplementary Table 3B.** The association of NRS2002 and PG-SGA with postoperative infection (In the cohort which the stage of cancer and previous treatment was available in CRC)

|  | | OR (95%CI) | | | *P* for trend | Per 1–SD |
| --- | --- | --- | --- | --- | --- | --- |
|  |  | Category 1 | Category 2 | Category 3 |  |  |
| NRS2002^†^ | No. of cases | 190 | 240 | 27 |  |  |
|  | Not adjusted | 1 | 1.18 (0.62–2.26) | 1.27 (0.35–4.67) | 0.585 | 1.25 (0.92–1.70) |
|  | No. of cases | 190 | 240 | 27 |  |  |
|  | Model 1 | 1 | 1.22 (0.64–2.33) | 1.18 (0.31–4.51) | 0.597 | 1.24 (0.91–1.69) |
|  | No. of cases | 190 | 240 | 27 |  |  |
|  | Model 2 | 1 | 1.17 (0.57–2.43) | 0.79 (0.19–3.28) | 0.979 | 1.09 (0.77–1.53) |
| PG-SGA^‡^ | No. of cases | 252 | 198 | 7 |  |  |
|  | Not adjusted | 1 | 4.38 (2.15–8.92) | 3.65 (0.40–33.01) | <0.001 | 1.90 (1.39–2.60) |
|  | No. of cases | 252 | 198 | 7 |  |  |
|  | Model 1 | 1 | 4.53 (2.21–9.26) | 3.75 (0.41–34.62) | <0.001 | 1.93 (1.40–2.65) |
|  | No. of cases | 252 | 198 | 7 |  |  |
|  | Model 2 | 1 | 3.86 (1.83–8.16) | 1.7 (0.15–19.91) | 0.001 | 1.73 (1.24–2.43) |

Model 1: Adjusted for age and gender. Model 2: Adjusted for age, gender, hypertension, diabetes, laparoscopy, albumin, prealbumin and BUN. CRC: colorectal cancer.

†: NRS2002 was classified as three categories by the criteria of “<3”, “3–4” and “≥5” score. ‡: PG-SGA was classified as three categories by the criteria of “A”, “B” and “C” grade.
